# Expanding on the ability of trivalent actinides to support microbial alcohol metabolism in evolved methylotrophic bacterium

**DOI:** 10.1038/s42004-025-01749-y

**Published:** 2025-11-21

**Authors:** Joshua J. Woods, Nathan M. Good, Alexia G. Cosby, Kirty Wadhawan, Jennifer N. Wacker, Alyssa N. Gaiser, N. Cecilia Martinez-Gomez, Rebecca J. Abergel

**Affiliations:** 1 Chemical Sciences Division, Lawrence Berkeley National Laboratory, Berkeley, CA USA; 2 Department of Plant and Microbial Biology, University of California, Berkeley, Berkeley, CA USA; 3 Department of Nuclear Engineering, University of California, Berkeley, Berkeley, CA USA; 4 Department of Chemistry, University of California, Berkeley, Berkeley, CA USA

**Keywords:** Metalloproteins, Biocatalysis, Chemical bonding

## Abstract

The 4 *f* elements, known as the lanthanides, have only recently been recognized as biologically essential metals. These elements can be found in the active site of certain alcohol dehydrogenase (ADH) enzymes, which are used by methylotrophic bacteria to metabolize simple alcohols such as methanol and ethanol. The 5 *f* elements, known as the actinides, often show similar chemical behaviour to the lanthanides and recent reports demonstrated that americium (Am) and curium (Cm) can replace lanthanides in certain ADHs to yield a catalytically competent enzyme and support bacterial life. In this work, we expand on these results to show the trivalent ions actinium (Ac^3+^), americium (Am^3+^), curium (Cm^3+^), berkelium (Bk^3+^), and californium (Cf^3+^) all support growth of an evolved strain of the model methylotroph *Methylobacterium extorquens* AM1. Further in vitro experiments using a reconstituted ethanol dehydrogenase enzyme confirm the ability of this enzyme to utilize trivalent actinide ions.

## Abstract (web summary)

Lanthanides have been identified as biologically essential metals, crucial in the active sites of alcohol dehydrogenase (ADH) enzymes in methylotrophic bacteria. Here, the authors demonstrate that trivalent actinide ions, including actinium, americium, curium, berkelium, and californium, can substitute lanthanides in ADHs and support bacterial growth.

## Introduction

The 4*f* elements, known as the lanthanides, comprise the 15 elements from lanthanum (La) to lutetium (Lu). Apart from promethium (Pm), which is radioactive, these elements are relatively abundant in the Earth’s crust. They were long believed unlikely to play a role in biological processes due to their poor bioavailability in the environment^1^. However, this view changed in 2011 when it was discovered that the methanol dehydrogenase enzyme known as XoxF, which is used by extremophile microbes to oxidize methanol to formaldehyde, can utilize the lighter lanthanides, from La up to samarium (Sm)^2–4^. Subsequent research into lanthanide biochemistry, which has been discussed thoroughly in several recent reviews^5–9^, has uncovered previously unknown lanthanide-dependent alcohol dehydrogenase (ADH) enzymes^10–14^ and bioaccumulation pathways for these metals^15–19^.

**Fig. 1:**
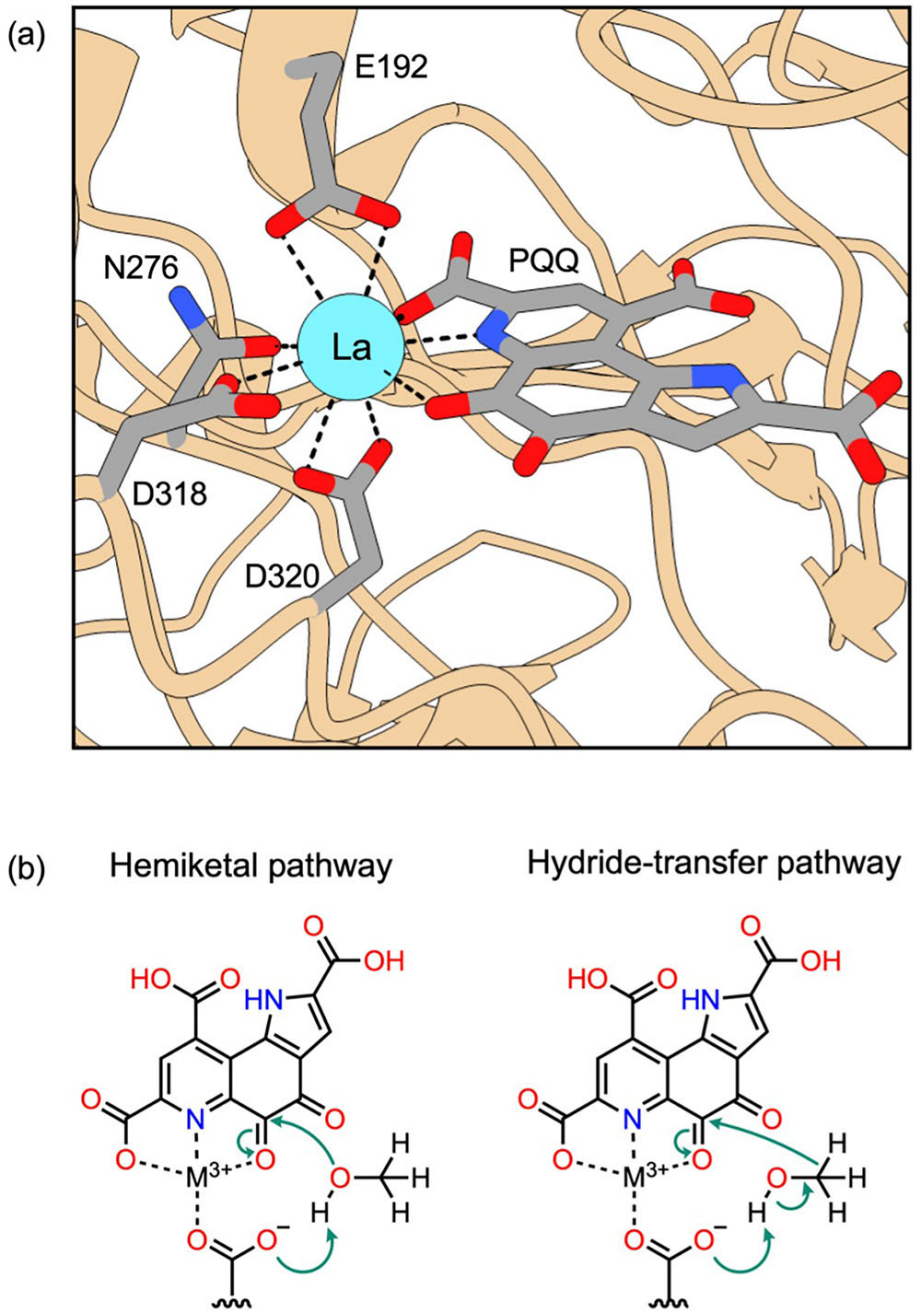
Metal-binding active site and proposed dehydrogenation mechanisms in lanthanide-dependent alcohol dehydrogenase enzymes. **a** Active site of methanol dehydrogenase XoxF bound to lanthanum (PDB 6OC6). **b** Schematic of the two hypothesized mechanisms of methanol dehydrogenation that take place in the active site of lanthanide-dependent ADH enzymes, where M^3+^ is the lanthanide cation.

Lanthanide-dependent ADH enzymes have become a topic of intense interest in recent years. Nanomolar concentrations of lanthanide ions in the environment trigger some bacteria to utilize lanthanide-dependent ADH enzymes for carbon assimilation and energy production instead of the calcium-dependent MxaFI homologs in a process that is known as the “lanthanide switch”^20^. The methylotrophic strain known as *Methylacidiphilum fumariolicum* SolV exclusively expresses the XoxF ADH enzyme and therefore rely solely on the environmental presence of lanthanides for their metabolism^15^. The MxaF and XoxF ADHs are classified as quinoproteins, meaning that their catalytic mechanisms require the presence of a quinone-containing group in the active site^21^. In the case of lanthanide-dependent ADH enzymes, pyrroloquinoline quinone (PQQ) is typically the redox-active cofactor (Fig. 1). An aspartate residue near the catalytic site is also required for enzyme activity^22^. The exact mechanism by which these lanthanide-dependent ADH enzymes function remains an open question, but two main pathways have been hypothesized in analogy to calcium-dependent ADH enzymes^23,24^. The first pathway involves an addition/elimination reaction with the formation of a hemiketal intermediate^25,26^, whereas the second involves a hydride transfer from the alcohol to PQQ (Fig. 1)^27^. Combined crystallographic and computational studies have suggested that the hydride transfer mechanism may be the operative mechanism^28–30^.

The 5*f* elements, known as the actinides, often show similar chemical behavior to their lanthanide counterparts. With the notable exceptions of thorium (Th) and uranium (U), most of these elements are not naturally occurring; they are typically produced in nuclear reactors or nuclear detonations and can be either accidentally or maliciously released into the environment. Given the similarities between the 4 *f* and 5*f* elements, actinides could be accumulated by microbial organisms and replace lanthanides in biochemical processes. Indeed, recent work has shown that americium (Am) and curium (Cm) can support methanol metabolism by methylotrophic bacteria and that lanthanide-binding proteins can coordinate actinide ions with high affinity^31–34^. These results hint at a possible pathway for mobilization of actinides in the environment and present a potential remediation strategy in the case of their dispersal into the environment^33,35^. In this work, we expand on these initial results and investigate the role of the metal ion in the alcohol metabolism of a genetically modified strain of *Methylobacterium extorquens* AM1 (AM1 = airborne methylotroph #1). We demonstrate that the trivalent ions of actinium (Ac^3+^), americium (Am^3+^), curium (Cm^3+^), berkelium (Bk^3+^), and californium (Cf^3+^) all support ADH activity in AM1, whereas actinides that have stable oxidation states other than +3 do not. Further in vitro experiments using a reconstituted ethanol dehydrogenase enzyme, known as ExaF^11^, confirm the catalytic activity of the ADH with trivalent actinide ions in the enzyme active site. The results reported herein provide further insight into microbe-actinide interactions and highlight potential pathways for actinide mobilization in the environment.

## Results and discussion

**Fig. 2:**
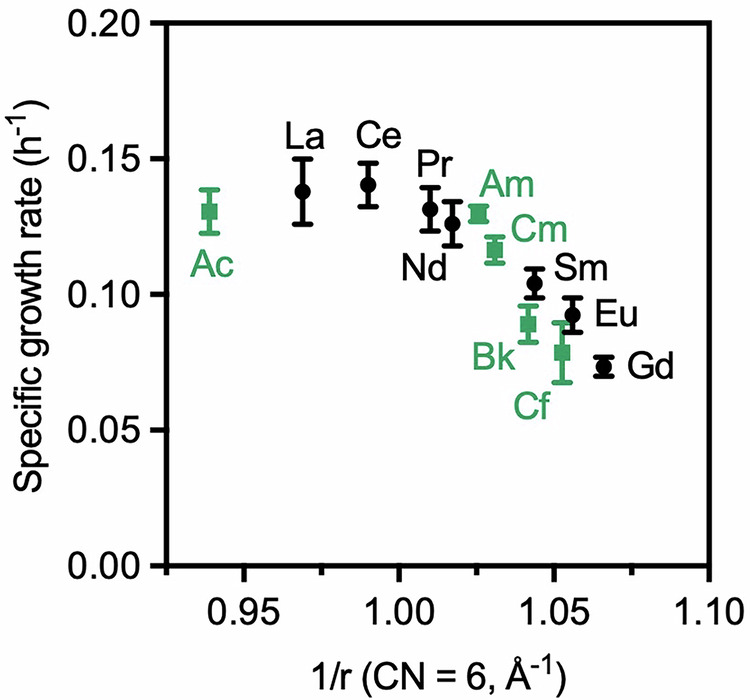
Compiled specific growth rate (h^−1^) of *evo*-HLn bacteria cultured in MP medium supplemented with 50 mM ethanol in the presence of trivalent lanthanides and actinides (0.5 µM) as a function of ionic radius. Ionic radii were taken from the literature^40,68^. Data were shown as the mean ± standard deviation of ten (lanthanides) or four (actinides) independent experiments. Representative growth curves and tabulated values for each element are given in the Supplementary Information.

For our investigations, we used a mutant strain of AM1 that lacks the genes encoding the large subunit of the calcium-dependent MDH pathway (*mxaF*) and the XoxF-type ADHs XoxF1 and XoxF2 to ensure that any observable alcohol oxidation would arise exclusively from the activity of the lanthanide-dependent ethanol dehydrogenase enzyme ExaF^11,20^. Importantly, the MDH-3 ∆*exa*F strain, in which the gene encoding ExaF is also removed, does not grow in the presence of lanthanides or alcohol^11^, meaning that any observed growth could be attributed to alcohol metabolism by ExaF. Specifically, we used the *evo*-HLn strain of AM1, a genetic variant that was evolved from AM1 MDH-3^20^, to accumulate gadolinium (Gd) and was found able to uptake and utilize Gd in contrast with ancestral MDH-3 and Δ*mxaF* strains^36^. We first validated the growth of *evo*-HLn bacteria in the presence of lanthanides. The bacteria were cultured in *Methylobacterium* PIPES (MP) medium^37^ supplemented with 50 mM ethanol and the desired metal ion (0.5 µM). The optical density of the culture at 600 nm (OD_600_) was monitored over time, and the corresponding specific growth rate was determined by fitting the data with the exponential growth equation. As shown in Fig. 2, bacterial growth was observed with all light lanthanides through Gd (except Pm, which was not tested due to a lack of availability), including Eu, which does not support the growth of ancestral MDH-3 and Δ*mxaF* strains^20,34^. Uptake experiments using ICP-MS confirmed that the metal ion was present inside the cells (Fig. S2). The *evo*-HLn bacteria showed the greatest accumulation of Gd^3+^, which was expected given that this strain was mutated specifically for hyperaccumulation of this ion^36^. The bacteria did not grow in the presence of the heavier lanthanides Tb and Dy (Fig. S1). Similar to what has been reported in previous studies, the bacterial growth rate decreases steadily when traversing the 4*f*-block (Fig. 2 and Table S1). This decrease in specific growth rate has been attributed to the decrease in the ionic radius of the lanthanide ion going across the series, which could affect metal transport within the organism or the incorporation of the metal into the active site of the enzyme. Subtle differences in ligand exchange rates and Lewis acidity may also influence the ability of the metal ion to promote alcohol oxidation^14,38,39^.

Following our initial experiments with lanthanide ions, we next explored the ability of the actinides to support bacterial metabolism. As with the lanthanides, bacterial cultures were incubated in MP medium containing 50 mM ethanol and 0.5 µM metal, and the change in OD_600_ was monitored over time. Notably, growth was observed for all cultures incubated with actinides that are most stable in the trivalent oxidation state in aqueous solution (Ac, Am – Cf). Uptake experiments using Am^3+^ as a representative trivalent actinide confirmed intracellular accumulation of the metal ion (Fig. S2). The actinides show a similar trend in bacterial growth as their lanthanide counterparts; the specific growth rate decreases with the ionic radius of the metal, with similar rates observed for metals that have similar ionic radii (Fig. 2 and S3 and Table S2). Notably, the specific growth rate of *evo*-HLn bacteria in the presence of Cm^3+^ is much higher than was reported for AM1 Δ*mxaF*^34^. It should be noted that the *evo*-HLn strain of bacteria used in this study was specifically evolved to accumulate large amounts of Gd^3+^^36^; therefore, it seems likely that the differences in microbial growth arise from differences in the metal uptake between the two strains rather than the actual metabolism process.

The observation that the relatively large Ac^3+^ ion enables growth of the AM1 *evo*-HLn bacteria was somewhat unexpected. This ion, with an ionic radius of 1.065 Å for the six-coordinated ion^40^, is the largest trivalent cation in the periodic table and exhibits the highest hydration number among the lanthanide and actinide elements in aqueous solution (10.0 ± 0.5)^41^. With this in mind, it was unclear if the ADH enzyme could incorporate such a large metal ion. Previous work has shown that the strontium (Sr^2+^) and barium (Ba^2+^) dications can replace calcium (Ca^2+^) in the active site of a Ca^2+^-dependent MDH isolated from *Methylobacterium extorquens* to yield a functional enzyme^42–44^. The enzyme loaded with Ba^2+^ displayed a lower activation energy for methanol oxidation compared to the Ca^2+^-containing MDH, but also possessed a much lower affinity for substrate (in this case MeOH), which was attributed to a change in the conformation of the enzyme active site^44^. It is possible that incorporation of Ac^3+^ into the active site of ExaF may similarly distort the enzyme and influence its substrate affinity, but we do not currently possess structural data to support this claim. Combined, these findings support the idea that Ac^3+^ could replace the lanthanide ions in the active site of ADH enzymes and support bacterial survival even though this ion is significantly larger than the metallic cations these organisms originally evolved to utilize for energy production.

The presence of Th, neptunium (Np), U, or plutonium (Pu) did not promote growth (Fig. S3), presumably because the corresponding ions that are most stable under the tested aqueous culture conditions (Th^4+^, [Np^V^O_2_]^+^, [U^VI^O_2_]^2+^, or Pu^4+^, respectively) are significantly different in charge or structure from the +3 actinide cations that fit the active site best. In fact, the uranyl ion has been shown to inhibit PQQ-dependent ethanol metabolism in *Pseudomonas aeruginosa*^45^. It should be noted that the reduction of Pu^4+^ to Pu^3+^ with hydroxylamine hydrochloride prior to addition to the culture medium did not promote bacterial growth either. We hypothesize that this result is due to the reoxidation of Pu^3+^ to Pu^4+^ in the culture media rather than some property of Pu that prevents bacterial growth^46^. However, we are unable to determine if Pu in any oxidation state is incorporated into the enzyme active site or if its binding yields a catalytically incompetent enzyme from this experiment. Additionally, it is possible that Th^4+^, [Np^V^O_2_]^+^, [U^VI^O_2_]^2+^, or Pu^4+^ undergo hydrolysis in the culture medium, preventing their accumulation by the bacteria and incorporation into the enzyme active site^47^.

**Fig. 3:**
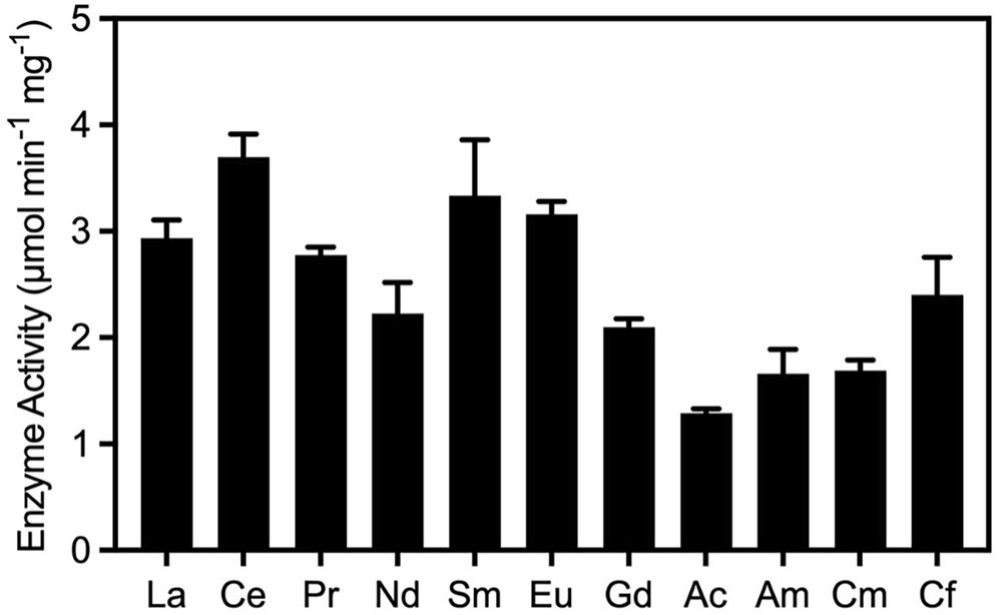
Enzymatic activity (µmol min^−1^ mg^−1^) of ethanol dehydrogenase enzyme ExaF reconstituted with different trivalent lanthanide and actinide ions as determined by a dye-coupled assay. Data were represented as the mean ± standard deviation of 3–5 independent experiments. Data for Bk^3+^ could not be collected due to the low availability of ^249^Bk at the time of these experiments.

To further explore the catalytic activity of the trivalent actinides in comparison to their lanthanide counterparts, we studied the ability of these ions to support catalytic turnover of ethanol by the ethanol dehydrogenase ExaF in vitro. The enzyme was isolated from AM1 *evo*-HLn under metal-free conditions and reconstituted in the presence of PQQ and the metal ion of interest. Ethanol oxidation was measured by monitoring the phenazine ethosulfate (PES)-mediated reduction of 2,6-dichlorophenolindophenoldichlorophenazine (DCPIP)^48^, and the determined enzymatic activities are displayed in Fig. 3.

Examination of the data for the actinide ions displays no clear trend with ionic radius, which is likely due to experimental limitations rather than the identity or size of the metal ion. Due to radiological safety concerns and the general instability of the reconstituted enzyme, we were unable to measure the binding affinity of ExaF for the actinide ions via traditional techniques, such as isothermal titration calorimetry (ITC)^49^. Similarly, we were not able to purify the enzyme from cells grown in the presence of each actinide to ensure that the activity measurements were performed with a fully functional enzyme. It is known that the metal content of the enzyme is directly related to its observed activity^50^. In an attempt to probe the effectiveness of our reconstitution protocol, we measured the molar ratio of metal to protein for enzyme samples reconstituted with all lanthanides and Am as a representative actinide (Fig. S4). As shown in Fig. S5, we observed ~20–40% loading of the enzyme with the desired metal. This result shows that not all the active sites in the enzyme were metalated, and the data presented in Fig. 3 should only be considered a qualitative assessment of the catalytic activity of the enzyme in the presence of different metal ions. Nonetheless, the results clearly demonstrate that Ac^3+^, Am^3+^, Cm^3+^, and Cf^3+^ are all capable of supporting ExaF-dependent ethanol oxidation. Further experiments with a more stable enzyme construct or, alternatively, an artificial metalloenzyme^51^ are currently underway in our laboratories and will aid in our understanding of the interaction between trivalent actinides and ADH enzymes.

## Conclusions

As noted above, many of the actinide elements are not normally found in nature and are instead typically dispersed into the biosphere and geosphere as a result of anthropogenic activities^52,53^. As such, it is critical to understand the interaction of these elements with the environment to support remediation efforts in the case of their uncontrolled release. The interaction between microbes and actinides has been a topic of intense research in the past decade and has highlighted how microbial organisms can significantly modify the solubility and mobility of these elements in environmental settings^33,54,55^. More recently, it has been suggested that microbes could be harnessed for the bioremediation of radiometals after their dispersal in the environment^35,56,57^.

In this study, we expanded on previous research by demonstrating the ability of Ac^3+^, Bk^3+^, and Cf^3+^ to support microbial alcohol oxidation, which has not been previously examined. The ability of these ions to support microbial life is somewhat surprising given the intense radiation produced during the radioactive decay of ^227^Ac, ^249^Bk, and ^249^Cf, although several bacterial strains in the genus *Methylobacterium* are known to be resistant to radiation^58^. In the bacterial growth experiments, the trivalent actinides behave similarly to their lanthanide counterparts, with the specific growth rate decreasing as the ionic radius of the metal decreases. Previous studies suggested that the cut-off for ionic size for Ln and An dependent growth of AM1 ∆*mxaF* bacteria was 0.98 Å^34^. Clearly, our results show that the *evo*-HLn strain, which only possesses the ethanol dehydrogenase ExaF, can utilize considerably smaller metal ions (Sm^3+^, Eu^3+^, Gd^3+^, Cm^3+^, Bk^3+^, and Cf^3+^) for alcohol oxidation. As discussed above, these differences likely stem from differences in metal uptake between bacterial strains rather than metabolism. This hypothesis is supported by the observation that AM1 *evo*-HLn grows in the presence of Cm^3+^, whereas AM1 ∆*mxaF* does not.

It should be noted that the activity of ExaF could be influenced by a multitude of factors apart from metal ion size. The actinides, especially the later actinides^59^, tend to display greater covalency in their bonding compared to the lanthanides^60^. Additionally, actinides differ significantly from lanthanides in their ligand exchange kinetics^61^ and absolute chemical hardness (*η*)^62,63^, which could alter the susceptibility of the PQQ cofactor to nucleophilic attack by the alcohol substrate. Similarly, distortion of the enzyme active site upon incorporation of the actinide ions could also influence the oxidation process. Ultimately, more detailed kinetic and structural studies will be required to precisely evaluate which properties of the metal ion contribute to the activity of this class of enzymes. The data presented herein highlights the ability of actinides to replace lanthanides in bacterial metabolism and suggests the potential of microbial organisms to be utilized as tools for environmental remediation or recycling of radioactive metals.

## Methods

*Caution! All of the isotopes used in this work (*^*227*^*Ac,* ^*232*^*Th*, ^*238*^*U,* ^*237*^*Np,* ^*238/240/241/242*^*Pu,* ^*241*^*Am,* ^*244/246/248*^*Cm,* ^*249*^*Bk, and* ^*249*^*Cf) pose significant health risks due to their spontaneous fission and/or the emission of ɑ-, β-, and γ-radiation. All radioactive materials were handled* *in a radiological laboratory equipped with portable α- and β-counting monitors, high-efficiency particulate air (HEPA) filtered fume hoods, and negative-pressure gloveboxes. Appropriate personal protective equipment, body and extremity dosimetry, and a Canberra Sirius 5PAB hand-and-foot personal contamination monitoring station at the entrance/exit of the laboratory were used to ensure researcher safety*.

### Radiological counting

Gamma spectra were acquired using an in-house-built high-purity germanium (HPGe) detector controlled using GammaVision software. The detector was calibrated using a multi-source standard containing ^241^Am, ^109^Cd, ^57^Co, ^139^Ce, ^203^Hg, ^113^Sn, ^85^Sr, ^137^Cs, and ^88^Y (Eckert & Ziegler Radiopharma Inc.). Quantification was performed using InterSpec v1.0.9 (National Technology and Engineering Solutions of Sandia, LLC.). The appropriate γ-ray emission lines and absolute γ-ray emission probabilities (*I*_γ_) were obtained from the National Nuclear Data Center NuDat 3.0 Database (https://www.nndc.bnl.gov/nudat3/). Liquid scintillation counting (LSC) was performed using a Perkin Elmer Tri-Carb 2910TR liquid scintillation counter controlled with QuantaSmart software (Perkin Elmer, Inc.) or a Wallac 1414 Guardian liquid scintillation counter controlled with WinSpectral software (Perkin Elmer, Inc.). LSC samples were prepared by diluting 1–2 µL of metal solution into 5 mL of Ultima Gold liquid scintillation cocktail (PerkinElmer, Inc.) and shaking to mix.

### Isotope stock solutions

The ^227^Ac, ^237^Np, ^242^Pu, ^241^Am, ^248^Cm, and ^249^Cf used in this study were purified as previously reported from legacy material available at Lawrence Berkeley National Lab^64–66^. The ^249^Bk used in this study was supplied by the US DOE Isotope Program. The ^227^Ac and ^249^Bk were used within 2 days of purification from their daughter products^66,67^ due to their relatively short half-lives (*t *= 21.772(3) y and 327.3(3) d respectively). All isotope stock solutions were prepared at a typical concentration of 10–20 µM in 0.1 M HCl, and the exact concentration of each stock was determined using gamma spectroscopy (^241^Am and ^249^Cf) or LSC (^227^Ac, ^237^Np, ^242^Pu, ^248^Cm, and ^249^Bk). A stock solution of Pu^3+^ was prepared by adding an excess of NH_2_OH·HCl to the Pu^4+^ solution and incubating at room temperature for 1 h. A stock solution of ^232^Th was prepared by diluting a standardized solution of Th(NO_3_)_4_ (BDH Chemicals) in 0.1 M HCl. A stock solution of ^238^U was prepared by diluting a standardized solution of UO_2_(NO_3_)_2_ (Sigma-Aldrich) in 0.1 M HCl.

Note—The Pu material used in this work consisted of 99.98% ^242^Pu, 0.02% ^240^Pu, <0.01% ^238^Pu, and «0.01% ^241^Pu by mass. The Cm material consisted of 97% ^248^Cm, 3% ^246^Cm, and <0.001% ^244^Cm by mass.

### MP medium

The *Methylobacterium* PIPES (MP) medium was prepared according to the literature^37^. This solution, optimized for culture of *Methylobacterium extorquens* AM1, contains 30 mM PIPES, 1.45 mM K_2_HPO_4_, 1.88 mM NaH_2_PO_4_, 0.5 mM MgCl_2_, 8 mM (NH_4_)_2_SO_4_, 20 µM CaCl_2_, 45.6 µM sodium citrate, 1.2 µM ZnSO_4_, 1 µM MnCl_2_, 18 µM FeSO_4_, 2 µM (NH_4_)_6_Mo_2_O_24_, 1 µM CuSO_4_, 2 µM CoCl_2_, and 0.33 µM Na_2_WO_4_. After adjusting its pH to 6.75 with KOH, the solution was sterilized by autoclaving at 121 °C for 45 min.

### Preparation of multicomponent (MC) buffer

This buffer was used for kinetic studies and contains 50 mM Tris-HCl, 50 mM Bis-Tris, 50 mM CHES, and 50 mM citric acid. After adjusting its pH to 9 with KOH, the solution was sterilized by autoclaving at 121 °C for 45 min.

### Bacterial strains and cultivation

The *evo*-HLn bacterial strain used in this study was reported previously^36^. Bacteria were inoculated on Bacto Agar plates as previously described. To prepare overnight cultures, colonies were harvested from the agar plate and inoculated into 2 mL MP medium supplemented with succinate (15 mM) and MeOH (50 mM). The tubes were incubated at 30 °C overnight before the bacteria were harvested by centrifugation. The resulting pellet was washed with MP medium and collected by centrifugation before being resuspended in fresh MP media. The suspensions were diluted to an OD_600_ = 0.1 prior to starting growth experiments.

### Bacterial growth assay

Bacterial cultures were inoculated into 96-well plates with OD = 0.1 at 600 nm in MP medium supplemented with 50 mM EtOH and 0.5 µM metal ion with a final volume of 200 µL per well. Control wells were prepared in the absence of a metal ion. The plate was incubated at 30 °C with shaking, and the OD at 600 nm was recorded every 30 min over 30 h. The specific growth rate was determined by fitting the exponential growth phase of the cultures using CurveFitter software (https://www.evolvedmicrobe.com/CurveFitter/). All data were reported as the mean ± standard deviation of ten (lanthanides) or four (actinides) independent growth experiments.

### Metal uptake assay

Bacteria were cultured in the presence of 0.5 µM lanthanide or actinide (Am^3+^) ion as described in the growth experiments. After 20 h, the cells were collected by centrifugation at 1000×*g* for 5 min. The resulting pellet was washed with 300 µL of fresh MP medium to remove any extracellular metal and was then resuspended in 100 uL of H_2_O. The cell membrane was mechanically disturbed by pipetting up and down several times. The cell debris was centrifuged at 1000×*g* for 5 min, and the supernatant was decanted and analyzed for metal content. The lanthanide concentrations were determined by ICP-MS, and the concentration of americium was determined using liquid scintillation counting. The amount of metal in each sample was normalized to the protein content of the sample, which was determined using the bicinchoninic acid (BCA) assay following the manufacturer’s instructions (Thermo Fisher). Each sample consisted of cells combined from three wells. Results are reported as the mean ± standard deviation of three independent trials.

### Enzyme isolation and purification

Unmetallated ExaF protein was produced via a genetic construct (pNG271) expressing ExaF from the strong mxa promoter (Pmxa) without the addition of lanthanides. Strain *evo*-HLn carrying pNG271 was grown in 2.8 L of MP medium without calcium. Methanol (50 mM) was added as the carbon and energy source, and kanamycin sulfate (50 µg/mL) was included for plasmid retention. Cultures were grown to an OD600 of 2.5–3.2 before cells were collected by centrifugation, flash frozen, and stored at −80 °C. The umetallated ExaF protein was purified as needed from these cultures following previously reported procedures^11^.

### Enzyme reconstitution and kinetics assay

A solution of enzyme (1 µM) was treated with equimolar amounts of the desired metal ion (in 0.01 M HCl) and PQQ. The solution was incubated in the dark for 2 h. To initiate the kinetic assay, the reconstituted enzyme solution was treated with methylamine hydrochloride (0.5 M in H_2_O, ~300,000 equivalents methylamine relative to enzyme) and 16 µL portions of the enzyme mixture were added to individual wells of a 96-well plate. The enzyme mixture was heated at 30 °C for ~1 min to equilibrate, after which 186 µL of reaction mixture (RM) containing 0.1 mM DCPIP, 1 mM PES, 15 mM EtOH, and 100 mM MC buffer. The final concentration of the enzyme in the assay well was 50 nM. Immediately after the addition of the RM, the progress of the reaction was monitored by measuring the decrease in absorbance at 600 nm as a function of time. Control wells were run in parallel where EtOH was substituted for H_2_O. The enzyme activity (µmol min^−1^ mg^−1^) was determined using the Michaelis–Menten equation using Prism GraphPad 6 (Graphpad Software). Results are reported as the average ± standard deviation of 3–4 independent experiments.

### Extent of metallation of the reconstituted enzyme

As above, a solution of enzyme (1 µM) was treated with equimolar amounts of the desired metal ion (in 0.01 M HCl) and PQQ. The solution was incubated in the dark for 2 h. The resulting solution was centrifuged using an Amicon Ultra 10 kDa MWCO spin filter following the manufacturer’s instructions. (Millipore Sigma). The lanthanide content of the reconstituted enzyme was determined by ICP-MS at the Laboratory for Environmental Analysis (Center of Applied Isotope Studies, University of Georgia). The actinide (Am) content of the reconstituted enzyme was determined by LSC. The protein content of each sample was determined using the BCA assay following the manufacturer’s instructions (Thermo Fisher). Results are reported as the mean ± standard deviation of three independent trials.

### Reporting summary

Further information on research design is available in the Nature Portfolio Reporting Summary linked to this article.

## Supplementary information

Supplementary Information

Reporting Summary

## Data Availability

All data supporting the findings of this study are available within the manuscript and the associated Supplementary Information file. Full experimental details, including representative growth curves, specific growth rates, intracellular metal concentrations, and additional figures and tables, are provided in the Supplementary Information.
