# Supplementary material for: Expanding on the ability of trivalent actinides to support microbial alcohol metabolism in evolved methylotrophic bacterium
Source: Commun Chem. 2025 Nov 21;8:367. doi: 10.1038/s42004-025-01749-y (PMC12639127; doi:10.1038/s42004-025-01749-y)
Supplement: Supplementary file 1 — Supplementary Information [file 42004_2025_1749_MOESM1_ESM.pdf]

Expanding on the Ability of Trivalent Actinides to Support Microbial Alcohol Metabolism in Evolved Methylotrophic Bacterium

Joshua J. Woods,[a†] Nathan M. Good,[b†] Alexia G. Cosby,[a] Kirty Wadhawan,[b] Jennifer N. Wacker,[a] Alyssa N. Gaiser,[a] N. Cecilia Martinez-Gomez,[b*] Rebecca J. Abergel,[a,c,d*]

[a]Chemical Sciences Division, Lawrence Berkeley National Laboratory, Berkeley, CA 94720, USA
[b]Department of Plant and Microbial Biology, University of California, Berkeley, Berkeley, CA 94720, USA
[c]Department of Nuclear Engineering, University of California, Berkeley, Berkeley, CA 94720, USA
[d]Department of Chemistry, University of California, Berkeley, Berkeley, CA 94720, USA

*abergel@berkeley.edu, cecimartinez@berkeley.edu
† These authors contributed equally.

Table of Contents

1. SUPPLEMENTARY FIGURES AND TABLES 2
2. SUPPLEMENTARY REFERENCES 6

1. SUPPLEMENTARY FIGURES AND TABLES

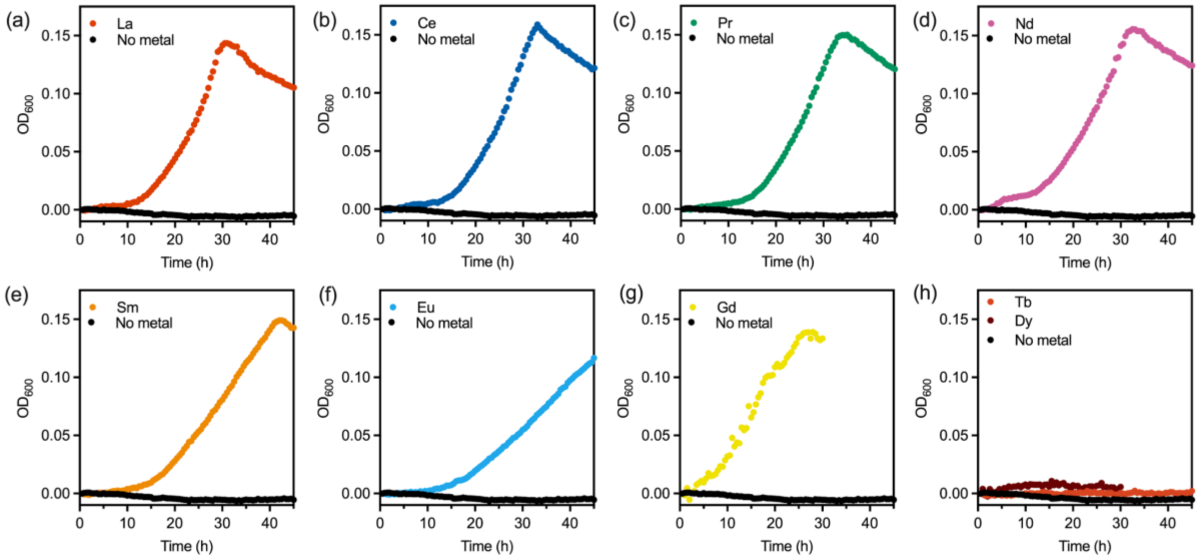

Figure S1. Representative growth curves of *evo*-HLn cultured in the absence or presence of each lanthanide ion (0.5 μM) in MP medium supplemented with 50 mM ethanol.

Table S1. Tabulated specific growth rates (h^{-1}) of *evo*-HLn bacteria cultured in the presence of trivalent lanthanides in MP medium supplemented with 50 mM ethanol. The number given in parentheses corresponds to the standard deviation of the last digit of the reported value (*n* = 10).

**Metal**	**Specific growth rate (h^{-1})**
La	0.138(12)
Ce	0.140(8)
Pr	0.131(8)
Nd	0.126(8)
Sm	0.104(5)
Eu	0.092(6)
Gd	0.073(4)
Tb	*No growth*
Dy	*No growth*

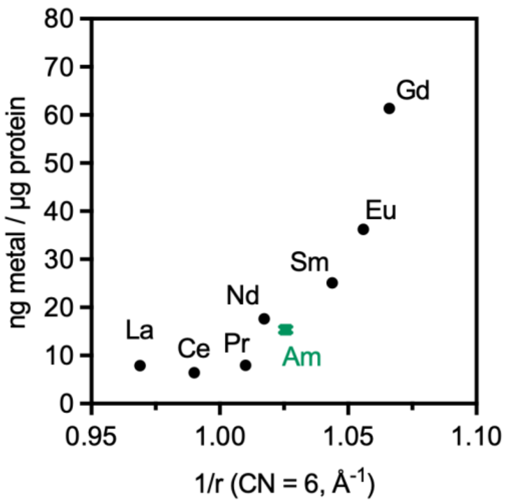

Figure S2. Cell uptake of lanthanide and actinide metal ions in *evo*-HLn bacteria. Cultures were treated with 0.5 µM metal ion and 50 mM ethanol and incubated for 20 h at 30 °C. The metal concentration was normalized to protein content, which was determined using the bicinchoninic acid assay. Data are represented as the mean ± standard deviation of three independent trials.

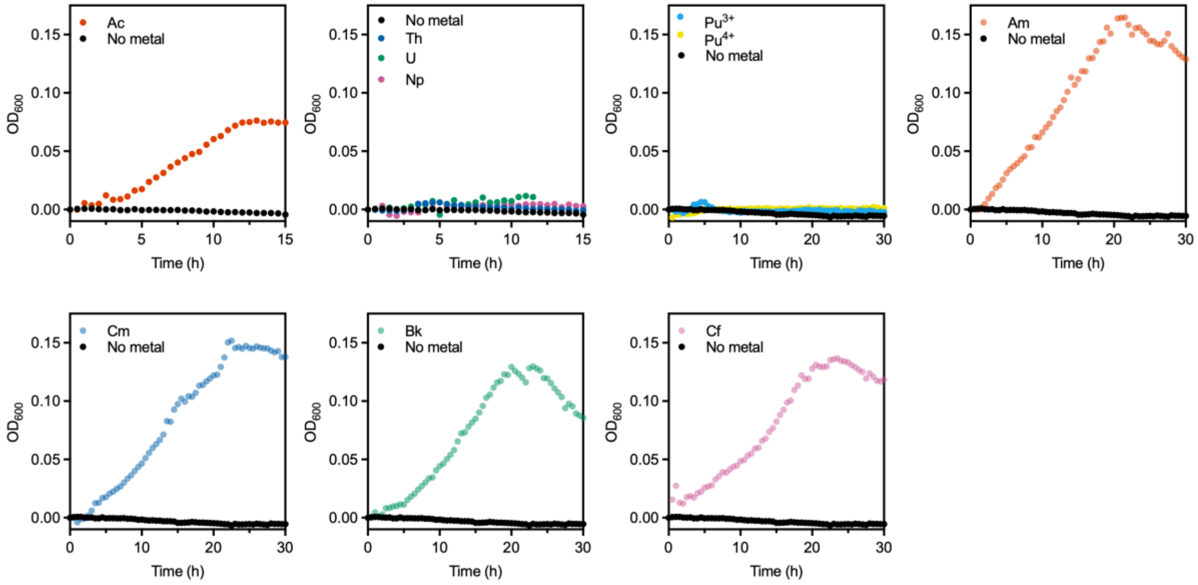

Figure S3. Representative growth curves of *evo*-HLn bacteria cultured in the absence or presence of each actinide ion (0.5 µM) in MP medium supplemented with 50 mM ethanol.

Table S2. Tabulated specific growth rates (h^{-1}) of *evo*-HLn bacteria cultured in the presence of actinides in MP medium supplemented with 50 mM ethanol. The number given in parentheses corresponds to the standard deviation of the last digit of the reported (n = 4).

Metal	Specific growth rate (h^{-1})
Ac	0.131(8)
Th	*No growth*
U	*No growth*
Np	*No growth*
Pu	*No growth*
Am	0.130(3)
Cm	0.116(5)
Bk	0.089(7)
Cf	0.079(11)

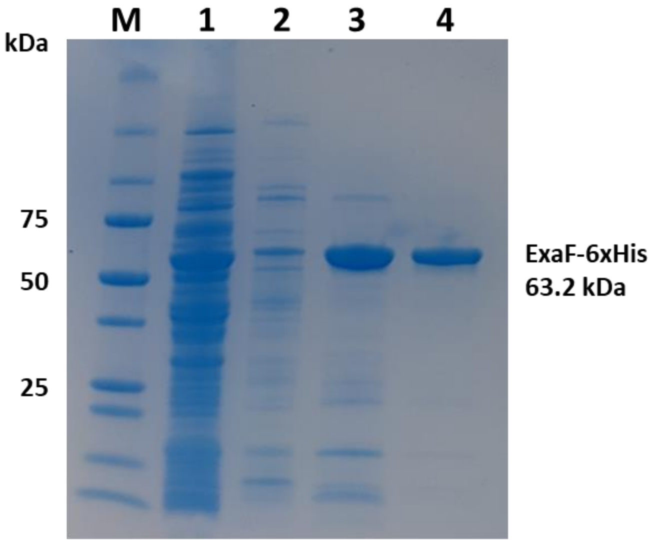

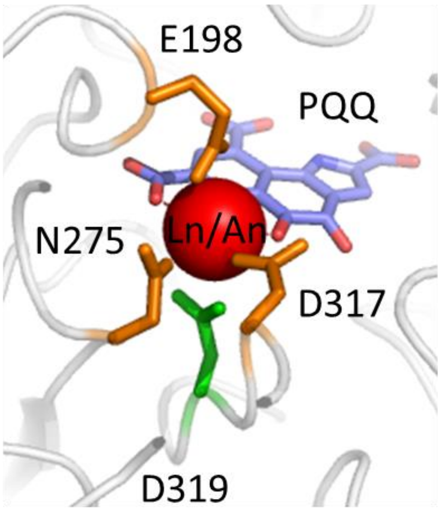

Figure S4. Unmetallated ExaF purification (left). M, peptide marker; 1, cell extract; 2, wash; 3, elution; 4, desalted elution. ExaF reconstitution with lanthanides and actinides (right). ExaF active site[1] modelled with coordinated generic Ln/An and PQQ.

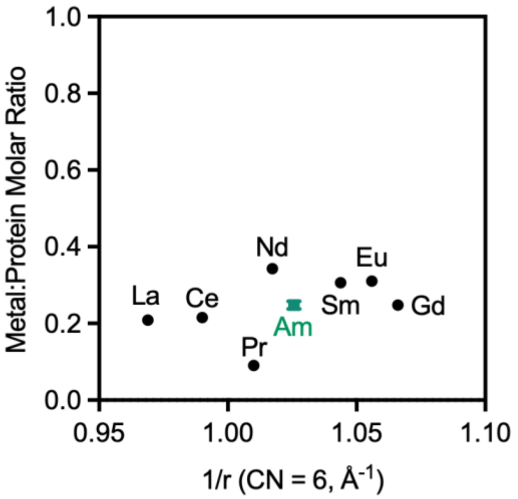

Figure S5. Molar ratio of metal to protein in ExaF reconstituted with lanthanides and Am.

2. SUPPLEMENTARY REFERENCES

1 Good, N. M. *et al.* Pyrroloquinoline Quinone Ethanol Dehydrogenase in *Methylobacterium extorquens* AM1 Extends Lanthanide-Dependent Metabolism to Multicarbon Substrates. *J. Bacteriol.* **198**, 3109-3118 (2016).
